# Unveiling the influence of caffeine on topiramate: metabolomic marker analysis using liquid chromatography-mass spectrometry

**DOI:** 10.3389/fmolb.2025.1549993

**Published:** 2025-06-17

**Authors:** Adrian Bartoszek, Ewa Paszkowska, Anna Kozub-Pędrak, Agata Sumara, Emilia Fornal

**Affiliations:** Department of Bioanalytics, Medical University of Lublin, Lublin, Poland

**Keywords:** caffeine, topiramate, zebrafish, epilepsy, seizure, zebrafish larvae, PTZ-induced seizure, metabolomics

## Abstract

**Background:**

Epilepsy affects approximately 70 million individuals globally, posing significant neurobiological and psychological challenges. Despite the availability of numerous antiepileptic treatments, one-third of patients remain resistant to therapy, with a limited understanding of caffeine (CAF) interactions with antiepileptic drugs such as topiramate (TPM). Zebrafish (*Danio rerio*), which share approximately 70% genetic homology with humans, represent a promising model for epilepsy research.

**Aim:**

To investigate the metabolomic alterations in zebrafish larvae subjected to a pentylenetetrazol (PTZ)-induced seizure model, specifically focusing on the effects of CAF and TPM.

**Methods:**

Four days after fertilization, zebrafish larvae were incubated for 18 h with different doses of TPM or a combination of CAF and TPM. Their locomotor activity was subsequently evaluated. Seizures were triggered by adding a PTZ solution to reach a final concentration of 20 mM. The identification of metabolites was carried out using liquid chromatography-tandem mass spectrometry (LC-MS/MS).

**Results:**

Our findings indicated lipid dysregulation, demonstrating increased levels of Lyso-PC, Lyso-PE, and Lyso-PAF in the epileptic larvae. Administration of TPM exacerbated lipid abnormalities, while CAF exhibited a stabilizing effect.

**Conclusion:**

The findings highlight the potential of metabolomic approaches in uncovering novel biomarkers, which could enhance the management and development of therapeutic strategies for epilepsy. Moreover, we highlight the complex interactions between CAF and antiepileptic medications. Our findings establish a foundation for further research to understand lipid metabolism and its relevance in epilepsy, potentially guiding future therapeutic strategies.

## 1 Introduction

Currently, approximately 70 million people worldwide are affected by epilepsy, a prevalent neurological disorder characterized by seizures that profoundly disrupt neurobiological functions and behavioral patterns (Stafstrom and Carmant, 2015). Individuals with epilepsy often face not only physical consequences, such as bruising and fractures resulting from seizures but also psychological challenges, including anxiety and depression (Stafstrom and Carmant, 2015). While symptomatic relief is offered through treatments such as antiepileptic drugs, surgical interventions, vagus nerve stimulation, or adherence to a ketogenic diet, a definitive cure for epilepsy remains elusive (Perucca, 2021). Moreover, around one-third of the epileptic populace exhibits resistance to the current pharmacological interventions despite the existence of over 30 antiepileptic drugs (AED) (Guery and Rheims, 2021). Despite ongoing advancements in understanding the molecular and cellular foundations of epilepsy, no preventive treatment currently exists to halt the onset of the disorder in individuals at risk. A major obstacle to rapid progress in this field is the complex and multifactorial nature of epilepsy, compounded by its variability (Stafstrom and Carmant, 2015). As previously noted, various medications are available for treating epilepsy, including drugs from several pharmacological classes. One of these is topiramate (TPM), which belongs to the anticonvulsant group and is widely used in managing seizures. Approved for monotherapy and adjunctive therapy, this treatment displays linear pharmacokinetics when administered alone. However, clearance is subject to influence by various factors, including age, renal function, and concurrent medication. It exerts its antiepileptic effects through multiple mechanisms, including inhibition of voltage-dependent sodium channels, enhancement of GABAergic transmission via GABA-A receptors, inhibition of AMPA/kainate glutamate receptors, and inhibition of carbonic anhydrase isoenzymes. According to its drug profile, TPM can interact with other substances that influence hepatic enzyme activity, especially those affecting CYP450 isoenzymes (Bae et al., 2016).

One of the most widely consumed stimulants worldwide is caffeine (CAF), a purine alkaloid. It is well established that CAF can interact with certain medications, potentially posing risks for individuals with epilepsy. Around 80% of the world’s population consumes beverages containing CAF, with North America and Europe having the highest *per capita* consumption (Quadra et al., 2020; Samoggia and Rezzaghi, 2021). The average daily CAF intake from coffee, tea, and soft drinks is approximately 250 mg per person, which is enough to have pharmacological effects (Fulgoni et al., 2015). Furthermore, CAF is an additive in a plethora of foods and beverages. Stimulants containing CAF, such as coffee and tea, not only influence epileptic seizures but also alter the efficacy of anticonvulsant medications, thereby complicating seizure management (Bauer and Sander, 2019).

The clinical data on the relationship between CAF and epileptic seizures is limited. Most of the existing understanding is derived from preclinical animal studies, a few clinical trials, and some case reports (Chrościńska-Krawczyk et al., 2011; van Koert et al., 2018). A recent study revealed that paraxanthine (PAR, the main CAF metabolite, which exerts similar to CAF action) and CAF levels stayed elevated and persisted overnight with daily intake. PAR levels remained higher even 24 h after CAF deprivation compared to the placebo (Lin et al., 2022). Current evidence indicates a complex interplay between epileptic seizures, CAF, and antiepileptic medications, complicating the development of definitive clinical guidelines for CAF consumption in individuals with epilepsy or those at risk. Preclinical studies suggest that while CAF can increase susceptibility to seizures, prolonged CAF intake could, in certain instances, provide some protective effects against them. Additionally, CAF has been shown to reduce the efficacy of several antiepileptic drugs, particularly TPM. CAF, primarily metabolized by CYP1A2, may influence the metabolism of co-administered drugs through enzyme induction or inhibition. Although CYP1A2 does not significantly metabolize TPM, indirect interactions may occur via shared pathways related to neurotransmitter modulation. One proposed mechanism of interaction involves CAF’s antagonistic effect on adenosine receptors, which may counteract TPM’s GABAergic and anti-glutamatergic actions, potentially reducing its anticonvulsant efficacy (Bartoszek and Fornal, 2025). The co-administration of CAF appears to modulate key metabolic pathways involved in TPM metabolism. Specifically, metabolomic markers indicate alterations in phase I and II metabolic enzymes, which could influence drug clearance and bioavailability (Kantae et al., 2016a). This aligns with existing research highlighting the role of metabolic enzyme modulation in drug interactions involving antiepileptic medications. The intricate relationship between CAF, seizure activity, and antiepileptic medications remains not fully understood, highlighting the need for further research in this area (Chrościńska-Krawczyk et al., 2011; van Koert et al., 2018).

One of the promising analytical method is metabolomics, which identifies various metabolites that indicate the organism’s current physiological state. By applying this comprehensive approach, dynamic alterations in metabolites can be systematically monitored across diverse biological processes, including development and disease progression. Untargeted metabolomics was applied for the study particularly as it allows for the comprehensive detection of a wide range of metabolites without prior assumptions about which ones may be affected. This unbiased approach is essential for uncovering unexpected metabolic changes associated with CAF and TPM administration in the epilepsy model. While most of the existing research in zebrafish studies has primarily focused on utilizing other “omics” technologies, it is worth noting that metabolomic data from zebrafish can be correlated with transcriptome data to uncover valuable insights into the novel biological functions of genes involved in metabolic processes (Soanes et al., 2011). A study on cyanide exposure found that metabolic changes in zebrafish larvae and humans are similar, indicating that metabolic changes are conserved across species (Nath et al., 2013). Consequently, investigating metabolic processes in zebrafish presents an exciting opportunity to explore such processes in epilepsy and potentially discover new biomarkers for controlling the diseases and creating guidelines for CAF intake. During seizures, when glucose availability is limited, the body may compensate by overproducing medium-chain fatty acids, lactate, and ketone bodies to meet the energy demands of neurons (Boison and Steinhäuser, 2018). This metabolic shift aligns with evidence suggesting that a ketogenic diet may offer protective effects in some types of epilepsy (Park et al., 2019).

In recent years, numerous metabolomic studies have been conducted involving epileptic patients, and various animal models, yet zebrafish have not been extensively studied in this context (Lai et al., 2022). These studies have primarily identified alterations in lipids, amino acids and organic acids as key metabolic changes associated with epilepsy-particularly glutamate, glutamine, 4-aminobutyric acid, choline, myo-inositol, and lactate. However, the levels of these metabolites showed inconsistent changes across different models (Lai et al., 2022). This inconsistency may result from the diverse seizure-inducing mechanisms, sampling time variations, and analytical platform differences. Moreover, no studies were conducted to assess metabolomic changes in epilepsy under CAF or CAF and TPM treatment. The study aimed to identify metabolite changes in response to CAF and TPM administration to understand the biological mechanisms through which CAF influences epilepsy in the zebrafish larvae pentylenetetrazol-induced seizure model. A simple, rapid and sensitive liquid chromatography–tandem mass spectrometry (LC–MS/MS) analytical method was employed for untargeted metabolite assessment in the zebrafish larvae.

## 2 Materials and methods

### 2.1 Animals

*Danio rerio* stocks of the wild type zebrafish strain (AB strain, Experimental Medicine Centre, Medical University of Lublin, Poland) were maintained at a temperature of 26°C–28.5°C in a controlled environment (pH ranging between 6.9 and 7.5; conductivity of 550–700; 14/10 h light/dark cycle). Embryos were bred under a standard light/day cycle in an E3 embryo medium in an incubator (IN 110 Memmert GmbH, Buechenbach, Germany). The 4 days post-fertilization (dpf) zebrafish larvae were used for the assays. After the experiment, larvae were immediately sacrificed by immersion in a solution of tricaine (15 μM). All experiments were conducted in accordance with the National Institute of Health Guidelines for the Care and Use of Laboratory Animals and the European Community Council Directive for the Care and Use of Laboratory Animals of 22 September 2010 (2010/63/EU). For the experiment with larvae up to 5 dpf, agreement with the Local Ethical Commission is not required (Dz. U. 2015 poz. 266).

### 2.2 Chemicals

Topiramate (TPM), caffeine (CAF), pentylenetetrazol (PTZ), ammonium bicarbonate, and formic acid (LC-MS grade) were obtained from Merck KGaA (Darmstadt, Germany). Acetonitrile and methanol (Optima® LC-MS grade) were supplied by Fisher Chemical (Waltham, MA, United States). A reference ion kit was obtained from Agilent (Santa Clara, United States). Tested compounds were dissolved in deionized water and diluted in E3 embryo medium (pH 7.1–7.3; 17.4 μM NaCl, 0.21 μM KCl, 0.12 μM MgSO_4_ and 0.18 μM Ca(NO_3_)_2_) to achieve a designated concentration.

### 2.3 Evaluation of locomotor behavior

Larvae were preincubated in 100 mL of E3 embryo medium or tested substances for 18 h in individual wells of a 96-well plate at 28°C. 10 larvae were used per treatment parameter and per experiment. The concentrations used in the study are 257.5 μM for CAF and 75 μM for TPM, based on our previous research (Bartoszek et al., 2023). After the preincubation, 100 mL of E3 embryo medium or 100 mL of a 40 mM PTZ solution was added to obtain a final concentration of 20 mM to evoke seizures (Afrikanova et al., 2013). Larvae were allowed to habituate for 5 min in a dark chamber of an automated tracking device (ZebraBox™ apparatus; Viewpoint, Lyon, France). The total locomotor activity was then quantified using ZebraLab™ software (Viewpoint, Lyon, France) (Afrikanova et al., 2013). Average movement or activity was expressed in ‘‘actinteg’’ units. The actinteg value of the ZebraLab™ software is defined as the sum of all image pixel changes detected during the time slice defined for the experiment (30 min).

### 2.4 Larvae treatment

Larvae were preincubated in 500 μL of E3 embryo medium or tested substances for 18 h in a 48-well plate (5 larvae per plate) at 28°C. After the preincubation, 250 μL of E3 embryo medium or 250 μL of a 60 mM PTZ solution was added to obtain a final concentration of 20 mM to evoke seizures. Larvae were allowed to incubate for 30 min with PTZ.

### 2.5 Sample preparation

Larvae were washed with E3 embryo medium three times, placed in a tube (20 larvae per sample), and homogenized by sonication (4 × 5 s) in 150 μL 100 mM NH_4_HCO_3_. Then samples were incubated on ice for 15 min and centrifuged (15,000 g, 15 min, 4°C). 100 μL of supernatant was mixed with methanol: ethanol solution (1:1) to obtain a 1:3 ratio, respectively, and vortexed for 30 s. After 15 min incubation at 20°C and centrifugation (15,000 g, 10 min, 4°C), 250 μL of supernatant was placed in a chromatography vial.

### 2.6 LC–MS/MS analysis

Chromatographic separation was conducted using Agilent Technology 1290 Infinity series high-performance liquid chromatograph connected to an accurate mass quadrupole time-of-flight 6550 iFunnel Q-TOF mass spectrometer equipped with a Jet Stream ion source (Agilent Technology, Santa Clara, CA, United States). The chromatographic separation was carried out using an Agilent Zorbax Extend-C18 rapid resolution HT (2.1 × 100 mm 1.8 μm) column with a gradient mixture of 0.1% formic acid in water (A) and 0.1% formic acid in acetonitrile (B) at the flow rate of 0.4 mL/min. The gradient program was as follows: 0–25 min, 3%–95% B, 25–30 min, 95% B. The injection volume was 10 µL and the column temperature was maintained at 45°C. The MS ion source operating conditions were optimized as follows: drying gas temperature 225°C, drying gas flow 12 L/min, nebulizer pressure 50 psi, and capillary voltage 1,000 V. The reference ions of *m/z* 121.0509 and 922.0098 were used for internal mass correction. The spectra were collected in positive ion mode (ESI+). Data acquisition was first performed in a scan mode, followed by a fragmentation mode (20 and 40 eV). During the acquisition of the fragmented spectra, chromatographic separation and ion source parameters were identical as in the scan spectra acquisition. Agilent Mass Hunter software versions B.09.00 and B.10.00 were used for data acquisition and data analysis, respectively.

### 2.7 Statistical analysis

Statistical analyses, regarding locomotor behavior, were performed by GraphPad Prism 8 (GraphPad Software, San Diego, CA, United States). The assumptions of normality and homogeneity of variances were verified using the Shapiro–Wilk test and Levene’s test, respectively. For comparison, data were analyzed using analysis of variance (one-way or two-way ANOVA). One-way ANOVA was followed by the Tukey’s test (*post hoc* test). In the case of two-way ANOVA, Bonferroni’s test was used as a *post hoc* test. The confidence limit of *p* < 0.05 was considered statistically significant. Data are presented as mean ± standard deviation (SD). Zebrafish larvae were randomly allocated to experimental groups. The experiments were performed in triplicate.

The raw MS data files were processed by Mass Hunter Qualitative B.10.00 software using the Find molecular feature algorithm and then imported into Mass Profiler Professional 15.1 for data processing (normalization, mass and retention time alignments, and mass profiling). Differential features were identified based on mass and fragmentation analysis using METLIN (http://metlin.scripps.edu/). Only identified compounds were subjected to further analysis.

Principal component analysis (PCA) and orthogonal last square discriminant analysis (OPLS-DA) were performed on aligned data sets using SIMCA software version 16.1 (Umetrics, MKS Instruments Inc.). Fold change cut-offs of 1.2 were applied (linear). Compounds altered with a *p* < 0.05 and exhibited >1.2 or <0.8 fold change between groups were classified as “changing” compounds unless otherwise stated in the text.

## 3 Results

### 3.1 The influence of caffeine and topiramate on larval locomotor activity

**FIGURE 1:**
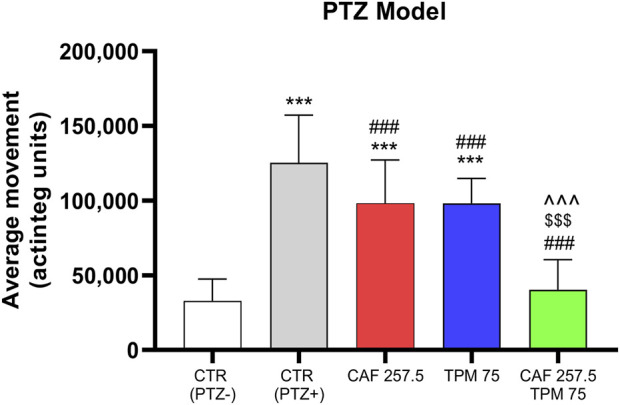
Effect of caffeine (CAF) and topiramate (TPM) on average larval locomotor movement in pentylenetetrazol (PTZ)-induced seizure model; TPM concentration applied: 75 μM; CAF concentration applied: 257.5 μM; ***p < 0.001, as compared to control (CTR, PTZ-); ###p < 0.001 as compared to control (CTR, PTZ+); ^^^ p < 0.001 as compared to CAF 257.5 μM; $$$ p < 0.001 as compared to TPM 75.

Firstly, the average movement of Zebrafish larvae, expressed in ‘‘actinteg’’ units, under the influence of CAF and TPM was assessed in the PTZ-induced seizure model. We have found that both 257.5 μM CAF and 75 μM TPM administrated alone significantly reduced the larvae locomotor activity compared to the PTZ group, but not to the control level. When administrating together, the movement was suppressed to the control level and was significantly different from both drugs administrated separately (Figure 1).

In a study conducted by Steenbergen et al 6 dpf larvae were treated with CAF (437.7 μM) for 7 min followed by a rapid washout, which did not alter the total swim distance (Steenbergen et al., 2011). In another study carried out in 7 dpf, 4 h treatment (437.7 μM) caused a significant reduction in the traveled distance, and the suppression was more pronounced after 24 h of exposure (Cruz et al., 2017). Confirming results were observed in 7 dpf larvae exposed for 2 h (acute exposure) before analysis and kept in the same solutions during the assay (Richendrfer et al., 2012). They found that 51.5 μM CAF did not change the swimming speed, while 515 μM suppressed it significantly (Richendrfer et al., 2012). It was also found that incubation 90 min post-fertilization significantly reduces distance at 120–168 h post-fertilization, especially at dark periods (Oliveira de Farias et al., 2021).

In a similar study, incubating 6 dpf larvae for 24 h with TPM in doses 1, 3, and 10 mM suppressed the locomotor activity compared to PTZ group in all three doses (Berghmans et al., 2007). TPM inhibited seizure-like behavior in 7 dpf larvae after 18 h incubation (200 μM), but failed to inhibit total seizure duration in the electroencephalogram assay (Afrikanova et al., 2013). Milder et al. carried out an experiment involving a 24-h incubation period and acute exposure to TPM (200 μM), noting a significant decrease in locomotor activity in 7-day post-fertilization (dpf) zebrafish, in comparison to the PTZ group (Milder et al., 2022). In the PTZ-induced seizure model (10 mM), the total movement for both the TPM pretreatment and the acute exposure groups was significantly lower than the controls. In contrast to Afrikanova et al. who faced discrepancies in locomotor and electroencephalogram assay (Afrikanova et al., 2013), they found that TPM reduced both behavior and neural activity, assessed by electroencephalogram and GCaMP studies (Milder et al., 2022).

Our findings present that TPM (75 μM) significantly protected larvae against the PTZ, but did not suppress the activity to the control level, which is in line with findings among 7 dpf larvae (180 μM, 18 h exposure) (Afrikanova et al., 2013). In 7 dpf larvae, 24 h TPM exposure (200 μM) decreased the activity even to the control level (Milder et al., 2022).

The number of studies regarding the interaction between CAF and TPM is limited. CAF at doses of 119 μM and 237.9 μM, given acutely or chronically, increased the amount of TPM, vital to protecting 50% of the mice (ED50) against maximal electroshock (MES)-induced seizure model (Chrościńska-Krawczyk et al., 2016). In contrast, CAF in lower doses (29.4 and 59.2 μM) did not affect the anticonvulsant action of TPM. In rats, CAF at doses 119 μM and 237.9 μM significantly enhanced the ED50 value for TPM, both when administrated chronically or acutely in MES-induced convulsions (van Koert et al., 2018).

Based on the results, we have concluded that these concentrations are optimal for further metabolomic assessment.

### 3.2 Zebrafish larvae analysis using pattern recognition

Firstly, as the initial step for sample differentiation, the PCA was performed for PTZ-treated groups to visualize data structure. The three-component model was obtained (*R* ^2^ = 0.897 and a Q^2^ = 0.735). Clear separation of zebrafish treated with CAF was observed in the first component, and separation of zebrafish treated with TPM was observed in the second component, which indicated a high potential of classification and discriminating power of studied groups based on metabolite profile. Thus, supervised pattern recognition analysis - OPLS-DA (orthogonal partial least squares discriminant analysis) was further applied to understand the differences between the groups.

**FIGURE 2:**
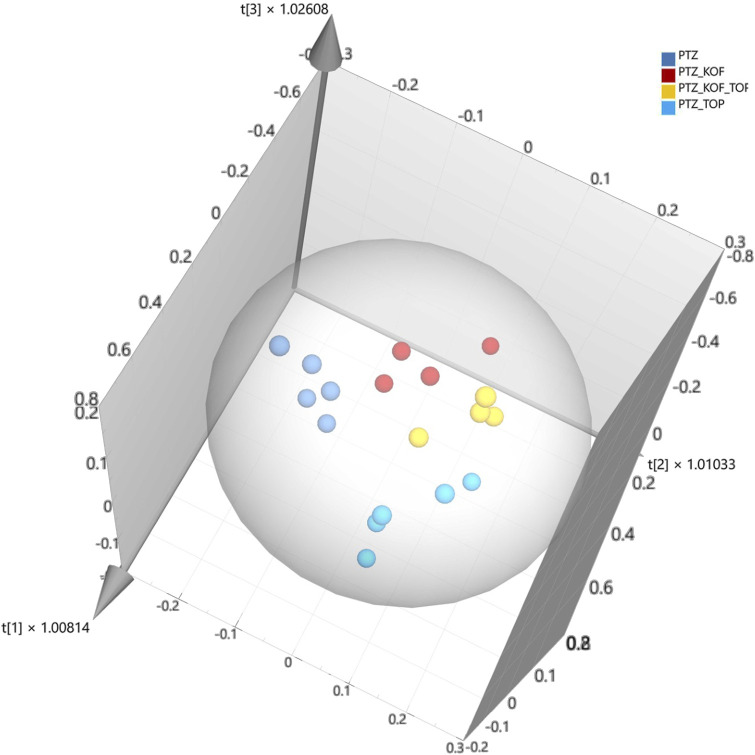
Score plot of the OPLS-DA model for experimental groups. Caffeine (CAF), topiramate (TPM), pentylentetrazol (PTZ); Scaled proportionally to R2X, R2X [1] = 0.733, R2X [2] = 0.119, R2X [3] = 0.0442, Ellipse: Hotelling’s T2 (95%); R2Y = 0.696, Q2 = 0.534.

The OPLS-DA model with the three predictive components was created. The model presents decent ability to explain and predict samples classification (R^2^Y = 0.696, Q^2^ = 0.534). Clear discrimination of the experimental groups is observed, the score plot is presented in Figure 2.

The variable importance for projections (VIP) value analysis was performed to identify metabolites, which play an important role in sample classification. Based on the analysis, we found that all 16 metabolites have a VIP value higher than 0.5 and 7 larger than 1, which is assumed to be the most influential value for this model (Table 1). The interval between 0.5 and 1 is a gray area, where the importance level depends on the size of the data set. It is not recommended to indiscriminately eliminate all variables below a value of 1. Instead, it is advisable to selectively exclude variables with the lowest VIP values (Andersen and Bro, 2010).

**TABLE 1:** Potential metabolites for differentiation of experimental groups obtained from VIP analysis for all four PTZ-treated groups.

No.	Metabolites	VIP value	VIP Standard error
1	Propionylcarnitine	1.71	0.42
2	9-cis/13-cis-retinal	1.26	0.40
3	C16 Lyso PAF	1.14	0.42
3	17:0 Lyso-PE	1.07	0.15
4	PE (P-18:0)	1.06	0.19
5	PE (P-16:0)	1.05	0.12
6	20:4 Lyso-PE	1.02	0.16
7	18:0 Lyso-PE	1.00	0.09
8	18:1 Lyso-PC	0.94	0.10
9	20:4 Lyso-PC	0.93	0.19
10	16:0 Lyso-PC	0.91	0.19
11	22:6 Lyso-PC	0.90	0.17
12	Lyso-PAF C-16	0.87	0.12
13	17:0 Lyso-PC	0.86	0.27
14	Inosine	0.76	0.11
15	Guanosine	0.68	0.75
16	Histidine	0.57	0.71

**FIGURE 3:**
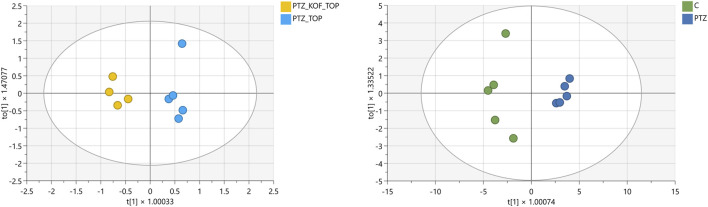
Score plot of the OPLS-DA model for experimental groups. Caffeine (CAF), topiramate (TPM), pentylentetrazol (PTZ); Scaled proportionally to R2X, R2X [1] = 0.429, R2Xo [1] = 0.391 and R2X, R2X [1] = 0.731, R2Xo [1] = 0.137, respectively. Ellipse: Hotelling’s T2 (95%).

Then OPLS-DA modelling was performed separately for CTR and PTZ groups, and PTZ_TPM and PTZ_CAF_TPM groups. The obtained models have good explanation and prediction performance (R^2^Y = 0.951, Q^2^ = 0.872, R^2^Y = 0.959, Q^2^ = 0.807, respectively). Clear classification and discrimination of the experimental groups were observed (Figure 3). VIP analysis revealed 16 metabolites with VIP values higher than 0.5 and 12 larger than 1 for the first model, and 17 and 12 metabolites for the latter, respectively (Tables 2,3).

**TABLE 2:** Potential metabolites for differentiation of experimental groups obtained from VIP analysis between CTR and PTZ groups.

No.	Metabolites	VIP value	VIP Standard error
1	16:0 Lyso-PC	1.11	0.16
2	22:6 Lyso-PC	1.10	0.15
3	18:1 Lyso-PC	1.10	0.11
3	20:4 Lyso-PC	1.10	0.18
4	PE (P-18:0)	1.08	0.16
5	18:0 Lyso-PE	1.08	0.15
6	Lyso-PAF C-16	1.08	0.36
7	17:0 Lyso-PC	1.07	0.36
8	PE (P-16:0)	1.07	0.18
9	17:0 Lyso-PE	1.07	0.16
10	20:4 Lyso-PE	1.06	0.18
11	Guanosine	1.04	0.25
12	C16 Lyso PAF	0.97	0.67
13	Inosine	0.90	0.74
14	Histidine	0.77	0.82
15	Propionylcarnitine	0.70	0.92
16	9-cis/13-cis-retinal	0.36	0.65

**TABLE 3:** Potential metabolites for differentiation of experimental groups obtained from VIP analysis, between PTZ_TPM and PTZ_CAF_TPM groups.

No.	Metabolites	VIP value	VIP Standard error
1	Propionylcarnitine	1.12	0.65
2	PE (P-16:0)	1.08	0.27
3	PE (P-18:0)	1.07	0.27
4	17:0 Lyso-PE	1.07	0.24
5	18:1 Lyso-PC	1.07	0.21
6	Lyso-PAF C-16	1.05	0.23
7	20:4 Lyso-PE	1.04	0.29
8	16:0 Lyso-PC	1.03	0.29
9	18:0 Lyso-PE	1.03	0.31
10	Inosine	1.02	0.50
11	22:6 Lyso-PC	1.00	0.30
12	20:4 Lyso-PC	1.00	0.29
13	Histidine	0.98	0.89
14	17:0 Lyso-PC	0.97	0.35
15	9-cis/13-cis-retinal	0.83	1.21
16	C16 Lyso PAF	0.70	0.66
17	Guanosine	0.59	0.31

Finally, we performed a t-test, resulting in metabolites altered with a *p* < 0.05 and exhibited fold change >1.2 or <0.8 between investigated groups (Table 4; Figure 3). Our study identified substantial metabolite dysregulations in PTZ-induced seizures in Zebrafish larvae. Notably, the most affected metabolites were lipids (Lyso-PC, Lyso-PE, and Lyso-PAF).

**TABLE 4:** Metabolites significantly changed (*p* < 0.05 and fold change <0.80 or >1.20) among the experimental groups. Caffeine (CAF), topiramate (TPM), pentylentetrazol (PTZ), fold change (FC).

Compound	CTR Vs. PTZ	PTZ Vs. PTZ_CAF	PTZ Vs. PTZ_TPM_CAF	PTZ Vs. PTZ_TPM	PTZ_CAF Vs. PTZ_TPM_CAF	PTZ_CAF Vs. PTZ_TPM	PTZ_TPM Vs. PTZ_TPM_CAF
	FC						
Inosine	0.64					1.41	
Guanosine	0.53						
Propionylcarnitine			1.64	2.26	1.67	2.31	0.72
2-Methylbutyroylcarnitine	0.66				1.52	1.49	
Histidine							
9-cis/13-cis-retinal	0.76						
17:0 Lyso-PC	0.80	0.77				1.46	
22:6 Lyso-PC		0.78				1.53	
16:0 Lyso-PC		0.73				1.58	
C16 Lyso-PAF	0.74	0.77				1.48	
18:1 Lyso-PC		0.69				1.67	
17:0 Lyso-PE	0.78	0.70	0.79			1.55	
PE (P-18:0)	0.78	0.66	0.79			1.62	
20:4 Lyso-PE		0.74				1.62	
PE (P-16:0)		0.65	0.80			1.68	
18:0 Lyso-PE		0.68				1.58	
20:4 Lyso-PC		0.74				1.61	

## 4 Discussion

Epilepsy encompasses a diverse range of neurological disorders, manifesting in various forms, making it challenging to capture the disease’s complexity fully. Consequently, multiple animal models are necessary to gain a comprehensive insight into epilepsy. Over the past 2 decades, zebrafish (*D. rerio*) has emerged as a valuable *in vivo* model used across various stages of research. Zebrafish are particularly advantageous due to their 70% genetic homology with humans, small size, similar molecular and pathological features to humans, low maintenance cost compared to other animal models, and rapid development (Howe et al., 2013; Kantae et al., 2016b). Importantly, despite a non-mammalian model, the central nervous system of *D. rerio* exhibits a remarkable degree of homology with humans (Kozol et al., 2016). This study provides novel insights into the interaction between CAF and TPM, utilizing advanced liquid chromatography-mass spectrometry (LC-MS) for metabolomic analysis. Our findings suggest that CAF significantly alters zebrafish’s lipid profile, potentially impacting its metabolism and leading to disease progression and altering cell membrane profile. Moreover, the precise management of epilepsy likely requires a combination of multiple metabolites rather than reliance on a single molecule. In our study, significant dysregulation of metabolites was observed in PTZ-induced seizures in zebrafish larvae, with lipids being among the most prominently affected metabolite classes.

### 4.1 Metabolomic studies

The field of omic sciences encompasses the comprehensive investigation of the various molecular components that contribute to the function and survival of living organisms. This includes the analysis of genes (genomics), proteins (proteomics), lipids (lipidomics), and small molecules or metabolites (metabolomics), each of which provides critical insights into different layers of biological regulation and processes. These interconnected fields facilitate a deeper understanding of the molecular mechanisms that drive the development and survival of different species (Lay et al., 2006). Metabolomics, a powerful analytical approach involving high-throughput profiling, provides key insights into the interactions within biological systems. By examining small molecules, known as metabolites or the metabolome, typically smaller than 1500 Da, metabolomics offers a distinct perspective on the physiological state of an organism, capturing dynamic changes in metabolic processes (Feng et al., 2020; Sindelar and Patti, 2020).

### 4.2 Primary metabolites

#### 4.2.1 Lipids

One class of metabolites that can be detected using this analytical technique is lipids, which are categorized as primary metabolites. These essential biomolecules play fundamental roles in energy storage, membrane structure, and cellular signalling, making their identification crucial for understanding metabolic processes (Rizo et al., 2020). Lipid metabolism—which includes uptake, transport, synthesis, and degradation—is a complex process. The production and breakdown of lipids are controlled by various signaling pathways. Different pathways may regulate a single lipid depending on the tissue, cell type, and whether conditions are normal, disease-related, or influenced by treatment (Mattes, 2005). Disruptions in lipid metabolism can modify cell membrane composition, alter protein localization and function, and change gene expression and cellular behavior. These shifts are linked to the initiation and progression of various diseases (Mattes, 2005). One of lipid dependent diseases is epilepsy. Studies in various models of epilepsy have revealed significant alterations in brain lipid metabolism, indicating their potential role in the pathophysiology of epilepsy. Phospholipids are fundamental components of neural membranes and play a critical role in synaptic transmission and neuronal signaling by interacting with specific membrane proteins (Bazan, 2014). Polyunsaturated fatty acids (PUFAs) serve as the key structural and functional elements of these phospholipids. A deficiency in n-3 PUFAs has been associated with a range of neuropsychiatric disorders, including schizophrenia, depression, mood disorders, attention deficit/hyperactivity disorder, post-traumatic stress disorder, and Alzheimer-type dementia (Yonezawa et al., 2020). In contrast, elevated levels of saturated free fatty acids—such as stearic, lauric, and palmitic acids—are linked to neurodegenerative processes, including dementia, stroke, epilepsy, spinal cord injury, Parkinson’s disease, neuroinflammation, and Alzheimer’s disease (Osorio et al., 2020).

**FIGURE 4:**
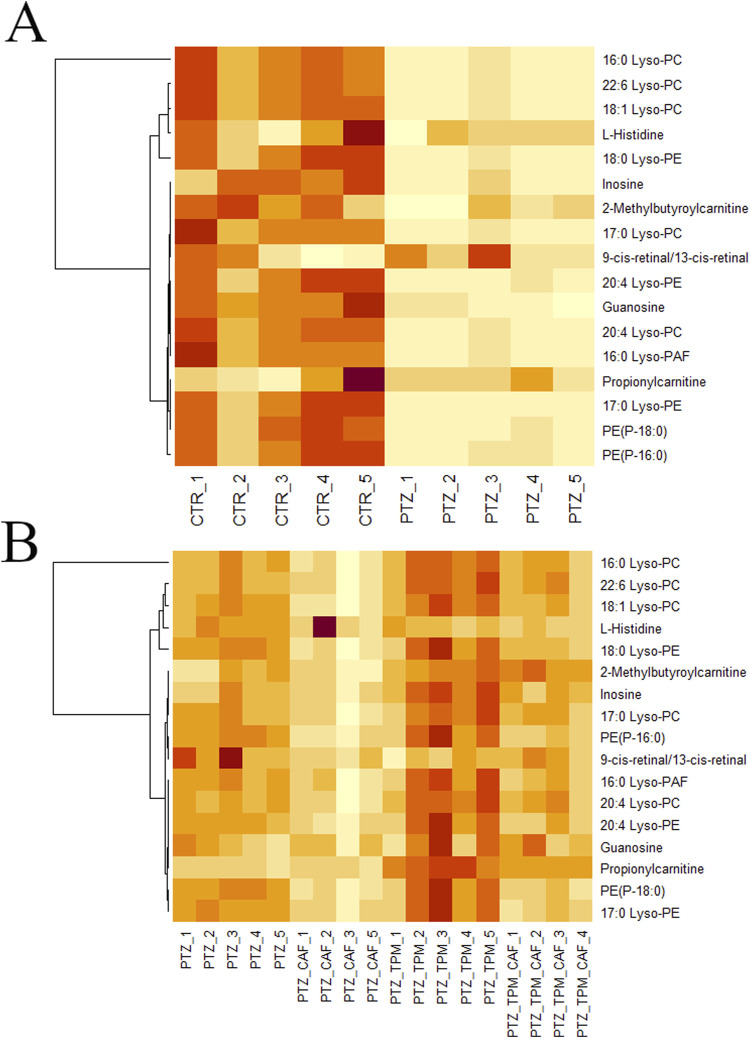
Heatmaps for metabolites among the experimental groups. **(A)** CTR and PTZ groups; **(B)** PTZ groups treated with CAF and TPM. Caffeine (CAF), topiramate (TPM), pentylentetrazol (PTZ).

Lerner et al. demonstrated that all examined mouse brain regions showed alterations in selected lipids, with lipidome varying in specific brain regions just 1 h after kainic acid (KA)-induced seizures (acute chemical kindling models) (Lerner et al., 2017). In the hypothalamus, nearly all the phospholipids (PLs) showed a statistically significant decrease (↓ PEs, PGs, PCs, PS, LPCs, LPAs, SM, hypothalamus) (Lerner et al., 2017). A year later same author presented that, in contrast to the previous study, the hippocampus exhibits the most changes in lipid levels at the acute seizure phase (↑ in PE, LPG, PG, PI, PS, LPA, PA, SM, LPC, PC, AEA, dorsal hippocampus) (Lerner et al., 2018). In the chronic KA model, the hippocampus exhibited an increase in Cers, GlcCers, ceramide phosphoinositols, DGs, and PCs, but in plasma, lower levels of PCs, TGs, DGs, and Vitamin D were observed (Heischmann et al., 2016). Among PTZ-induced chronic rat model, the brain exhibited an increase in PE and a reduction in GlcCers (Qiu et al., 2020). In Kv1.1-Knock out mice model of epilepsy, a decrease of DG, PA, PI, PE, and TG was observed in the brain samples (Johnson et al., 2020). In our study, the most affected metabolites were lipids. Epilepsy larvae exhibited a decrease in lipids (Lyso-PC, Lyso-PE; Lyso-PAF) compared to the control group (Figure 4A). Within epileptic groups, CAF significantly decreased lipids in animals compared to the PTZ group and larvae treated with TPM (Figure 4B). TPM alone does not affect lipids in our study. The altered metabolites belong to different lipid classes, primarily glycerophospholipids, lysophospholipids, carnitine derivatives, and retinoids, each with distinct physiological roles. A notable reduction in propionyl carnitine, a short-chain acylcarnitine, implies potential mitochondrial dysfunction or altered fatty acid metabolism (Mu et al., 2022). This metabolite serves as an intermediate in lipid oxidation and energy production; thus, their depletion may indicate reduced mitochondrial β-oxidation efficiency, leading to metabolic stress and energy deficits in the developing larvae (Adeva-Andany et al., 2019). Among the glycerophospholipids, a decline in lysophosphatidylcholine (Lyso-PC) and lysophosphatidylethanolamine (Lyso-PE) species, including 18:1 Lyso-PC, 20:4 Lyso-PC, 16:0 Lyso-PC, 22:6 Lyso-PC, 17:0 Lyso-PC, and 20:4 Lyso-PE, suggests disruptions in membrane remodeling and lipid signaling. Lysophospholipids are integral to cellular membrane dynamics and act as precursors for bioactive lipid mediators. Their reduction may compromise membrane integrity, affecting cell proliferation, differentiation, and overall tissue development (Wang and Tontonoz, 2019). Additionally, a decline in platelet-activating factor (PAF) derivatives, including C16 Lyso-PAF and Lyso-PAF C-16, suggests altered inflammatory and immune responses (Upton et al., 2022). Glycerophospholipids, including phosphoethanolamine (PE) and phosphatidylcholine (PC), are the predominant lipids in cell membranes, making up 25% of the brain’s dry weight.

They are essential for the physiological functions of neural cells (Palsdottir and Hunte, 2004; Zong et al., 2018). Disturbances in the homeostasis of glycerophospholipid species can impact endocytosis, exocytosis, membrane fusion, and neurotransmitter uptake and release (Farooqui et al., 2000). PE and PC undergo various transformations to produce choline. In rodent models, increased choline intake during gestation and early postnatal development has been demonstrated to protect the brain from epilepsy-related neurological damage, likely by reducing compensatory cell proliferation in the dentate gyrus (Wong-Goodrich et al., 2008), preventing the loss of GAD65 mRNA in the hippocampus (which encodes a form of the GABA-synthesizing enzyme) (Wong-Goodrich et al., 2008), and increasing the levels of trophic factors in the hippocampus (Ricceri et al., 2011). In patients with ischemic stroke, neurotransmitter levels are regulated by reversing changes in lipids such as PE, PC, and sphingomyelins (Wang et al., 2021). Future studies should aim to elucidate the regulatory mechanisms underlying these lipidomic shifts and their long-term impact on organismal physiology and development. Continuing, lipidomic studies of epileptic patients are scarce. Total triglycerides were reduced in the hippocampus of mesial temporal lobe epilepsy patients (n = 47) (Zhang et al., 2020). Patients (n = 3) treated with valproic acid exhibited an increase in TGs, SMs, PCs, and Cers and a reduction in PEs, DGs compared to control patients in plasma, assessed by HPLC TOF–MS (Li et al., 2019). Lipids (not specified by the authors) were also reduced in the post-mortem brain of epileptic patients (n = 15), assessed by DI/LC–MS/MS (Lalwani et al., 2020). Unfortunately, no studies were conducted in patients treated with TPM.

#### 4.2.2 Amino acids

The next significant changes pertain to amino acid components. In electrically induced seizures, decreased histidine levels (VIP = 1.1) in mice plasma were observed with the use of capillary electrophoresis coupled to mass spectrometry (Segers et al., 2020). Similar results were presented in rat plasma among the KA model of temporal lobe epilepsy (Heischmann et al., 2016). L-histidine, a precursor for histamine, is reported to have anticonvulsant activity in different rat seizure models (Borowicz et al., 2000). Moreover, it was presented to enhance the protective effects of carbamazepine and phenytoin (Kamiński et al., 2004). The action is mediated by H1 receptors. In the present study, histidine was found to be increased in the epilepsy model when compared to the control group. Neither CAF nor TPM affected this metabolite.

Amino acids and organic acids are the main types of metabolites reported to be altered in epilepsy. Despite alterations in alanine, aspartate, arginine, and glutamate levels and their metabolic pathways were observed in both animal (Shirayama et al., 2005; Yang et al., 2018) and human studies (Wei et al., 2012; Wang et al., 2016; Hanin et al., 2024) our study did not find these metabolites differentiating. Pathway changes including glutamine, citrate, asparagine, NAAG, and succinic acid semialdehyde may affect the essential glutamine–glutamate cycle between neurons and astrocytes, which could be a factor in causing hyperexcitability (Rho and Boison, 2022).

Another significant metabolite is guanosine, which was shown to have antiepileptic potential in a few mice (Schmidt et al., 2000) and rats (Kovács et al., 2015) models, however, Lakatos et al. presented that higher guanosine doses (100 mg/kg) have the opposite effect, by increasing the spike-wave discharges (SWDs) in rat absence epilepsy model (Lakatos et al., 2016). The possible mechanism involves binding to its G-protein coupled receptor and enhanced astrocytic glutamate uptake (Lakatos et al., 2016). Additionally, THY pretreatment protected rats against high guanosine dose (100 mg/kg) (Kovács et al., 2015). In our study guanosine was decreased in the epilepsy model compared to the control group. Neither CAF nor TPM affected this metabolite.

Guanosine can be also converted to Inosine, which increases SWD number (Kovács et al., 2015), protects mice from quinolinic acid-induced seizures (Schmidt et al., 2000), antagonizes CAF-induced seizures (Marangos et al., 1981), and increases the threshold of PTZ seizures (Skolnick et al., 1979). The assumed mechanism is mainly through inhibitory gamma-aminobutyric acid receptors (GABA A receptors) and adenosine receptors (Kovács et al., 2015). In our study inosine was increased in the epilepsy model compared to the control group. Larvae administrating CAF also exhibited an inosine increase compared to larvae treated with TPM.

#### 4.2.3 Other metabolites

Carnitine is crucial for β-oxidation by facilitating the transport of acyl groups from fatty acids across the mitochondrial membrane. Despite most of the substance is derived from diet, there is also an endogenous carnitine fraction. Its low level has been mainly associated with patients prescribed valproate (Okumura et al., 2021), but the study in rats presented the anticonvulsant properties of L-carnitine in PTZ-induced seizures (Hussein et al., 2018). The protective properties of carnitine are associated with antioxidant activity, anti-apoptotic effects, upregulation of neuroprotective heat shock protein, and suppression of autophagy (Hussein et al., 2018). We observed alterations in two carnitine derivatives. Propionylcarnitine levels increased in the group treated with TPM, but when co-administered with CAF, its levels decreased compared to TPM treatment alone. 2-Methylbutyroylcarnitine differentiated PTZ-treated larvae from a control group and those treated with CAF compared to TPM and CAF-TPM combination.

A ketogenic diet has demonstrated protective effects in epilepsy management, though careful monitoring of carnitine levels may be necessary in these patients to prevent potential deficiencies (Chu et al., 2024).

9-cis/13-cis-retinal are naturally occurring retinoids, vitamin A analogs. Vitamin A administrated chronically was shown to possess anti-epileptogenic activity in the PTZ-kindling model of epilepsy (Sayyah et al., 2005). In a recent review, possible mechanisms of action were assigned to the main vitamin A metabolite. Retinoic acid is a metabolite with antiepileptogenic properties that exerts its effects through multiple mechanisms. These include the modulation of gap junctions, neurotransmitter activity, long-term potentiation, and calcium channels, all of which contribute to its potential to influence seizure dynamics (Rosiles-Abonce et al., 2021). In our study, these metabolites were decreased in the epilepsy model compared to the control group. Neither CAF nor TPM affected this metabolite.

#### 4.2.4 Influence of TPM on lipid profile

TPM is an anticonvulsant drug used for the treatment of epilepsy, and migraine (Bae et al., 2016), but its off-label use includes essential tremors, alcohol disorders, and weight reduction (Wajid et al., 2023). To date, there are no metabolomic studies with patients administrating TPM, but a few assessed base lipid profiles. Switching 13 epilepsy patients from monotherapy with phenytoin or carbamazepine to monotherapy with TPM reduced all cholesterol fractions and triglycerides (TG) (Mintzer et al., 2012). On the other hand, in epileptic patients administrating at least two AED for 6 weeks (2 on TPM), no changes in lipid profiles were observed (TC, HDL, LDL, TG) (Sarecka-Hujar et al., 2022). Similar findings were observed in women with epilepsy on AED monotherapy (Kim and Lee, 2007) and 70 children on TPM monotherapy after 12 months of treatment (Franzoni et al., 2007). Patients administrating weak enzyme-inducing AED (including TPM) had significantly higher LDL levels compared to those receiving drugs with non-inducing enzymes, but other lipids fractions were not changed (Muller et al., 2023). Six months of TPM monotherapy in 33 premenopausal women with cryptogenic epilepsy resulted in HDL reduction (Oguz Genc et al., 2010).

Few studies have evaluated the lipid profiles of obese patients without epilepsy undergoing TPM treatment. In diabetic patients managed with diet or sulfonylurea, TPM therapy for 11 months resulted in weight loss and improved glycemic control, but did not alter TC, TG, HDL, LDL, or apolipoprotein B (Eliasson et al., 2007). In hypertension normolipidemic patients TPM reduced TG (Tonstad et al., 2005). However, in normolipidemic patients on metformin, no lipid changes were observed after 8 weeks of TPM treatment (Toplak et al., 2007). Similarly, a 6-month therapy in obese patients did not result in any significant lipid alterations (Bray et al., 2003).

#### 4.2.5 Influence of CAF on lipid profile

CAF consumption led to a rise in energy expenditure by about 13% and significantly boosted lipid turnover and oxidation. However, most lipid disposal (76%) occurs via nonoxidative pathways, with only 24% attributed to lipid oxidation (Acheson et al., 2004).

To date, no studies regarding metabolomics have investigated metabolomic changes in epilepsy under CAF treatment. However, research in rat brains has shown a decrease in both metabolites and lipid levels, with proteomic analysis indicating reduced energy metabolism due to lower levels of proteins involved in mitochondrial function (Paiva et al., 2024). Additionally, it has been reported that protein regulation-particularly those related to synaptic function remain altered even after a 2-week chronic CAF withdrawal, highlighting the long-lasting effect of CAF effect on neurons (Paiva et al., 2024).

##### 4.2.5.1 Pharmacokinetic and pharmacodynamic considerations

CAF’s impact on TPM metabolism raises important pharmacokinetic considerations. Enhanced metabolism may lead to sub-therapeutic levels of TPM, potentially compromising seizure control, while reduced clearance could increase the risk of adverse effects. These findings are consistent with studies indicating that CAF can significantly reduce the anticonvulsant activity of various antiseizure medications, including TPM, in animal models.

##### 4.2.5.2 Translating animal model to human

Although zebrafish (*D. rerio*) do not perfectly mimic human physiology, they offer a robust compromise between ethical imperatives and experimental efficacy. By adhering to the 3Rs principle, zebrafish reduce animal suffering while enabling high-throughput screening for toxicity, pharmacokinetics, pharmacodynamics, and drug interactions. Their small size, rapid development, and genetic similarity to humans make them an effective, cost-efficient model for early drug evaluation, despite inherent limitations. No model is flawless, yet zebrafish remain invaluable in bridging the gap between basic research and clinical application.

It was presented that the dynamics of seizures in zebrafish and humans are similar, which enhances the transferability of results from the animal to human clinical context (Jirsa et al., 2014).

With the use of paracetamol, it was also shown that *D. rerio* clearance correlate rationally well with higher vertebrae (Kantae et al., 2016b; van Wijk et al., 2019). Zebrafish larvae are intermediate solutions between *in vitro* and *in vivo* mammal studies, because of the experimental efficiency and the possibility to study whole vertebrate organisms while maintaining the 3 R’s rule.

Although using larvae for experiments offers several advantages, it necessitates a thorough understanding of how the developmental stages influence the features under investigation. A comprehensive understanding of larval development is pivotal for the effective design of studies and precise interpretation of observed results. CAF is metabolized in the liver through demethylation, which is predominantly catalyzed by the cytochrome P450 enzyme family, almost exclusively CYP1A2 (95%). This enzyme converts CAF to its primary metabolites, including paraxanthine (PAR), theobromine (THM), and theophylline (THY) (Chen et al., 2017). PAR is the most abundant (84%) and biologically active metabolite of CAF. It is responsible for most of CAF’s pharmacological effects, including its stimulant properties (Lin et al., 2022). According to prior studies, the development of the hepatic primordium in zebrafish initiates at 28 h post-fertilization (hpf), followed by hepatic outgrowth between 60 and 72 hpf, and by 120 hpf, the liver function, which includes CYP metabolism, is nearly fully formed (Tao and Peng, 2009). However, it was shown that CYP metabolic functions (especially CYP1A2) are similar to those of human CYP isoform even before full liver development, confirmed by both mRNA and metabolite assessment in 24–120 hpf larvae (Nawaji et al., 2020). Translating findings from animal studies to human contexts poses significant challenges, particularly due to differences in CAF metabolism across animals, humans, and animal models. Animal studies often employ CAF doses far exceeding the estimated equivalent average human CAF consumption. In humans, CAF plasma concentrations of 77.2 μM or higher typically stimulate the central nervous system and cardiovascular system. While life-threatening CAF consumption is rather associated with drugs containing this methylxanthine than caffeinated food or beverages, overdose cases have reported concentrations as high as 978.4 μM, with lethal intoxications documented at 1802.4 μM and 2919.8 μM (Kerrigan and Lindsey, 2005). Despite the promising insights provided by metabolomic studies, translating findings from zebrafish models to humans remains challenging. Environmental conditions during larval breeding, including temperature, photoperiod, water quality, nutritional status, and microbial exposure, may have influenced the observed metabolic profiles and introduced variability. Additionally, limitations inherent to current analytical platforms constrain the coverage of the full metabolome, potentially leading to the omission of relevant but undetected metabolites and thereby complicating data interpretation. However, the metabolic alterations observed in this study reflect potential targets for therapeutic intervention and highlight the need for further exploration into lipid metabolism and its role in epilepsy. Future research should focus on validating these findings in mammalian models and human studies, particularly in patients undergoing treatment with antiepileptic drugs like TPM, and exploring the broader metabolic impacts of CAF and other dietary factors in epilepsy.

## 5 Conclusion and future perspectives

While preclinical models have inherent limitations, our study highlights the value of metabolomic approaches in predicting clinically relevant drug-drug interactions. The cost-effectiveness and high sensitivity of LC-MS make it a powerful tool for early screening of metabolic interactions. Notably, the observed interplay between CAF and TPM may have significant clinical implications, given the widespread consumption of CAF. Although experimental studies suggest that CAF can attenuate the efficacy of certain antiseizure medications, clinical findings remain inconsistent, with some studies reporting no significant impact. This underscores the necessity for further clinical investigations to fully elucidate the implications of CAF intake in patients undergoing TPM therapy.

Our findings also confirm that CAF exhibits extensive interactions across study groups. As a component of foods and beverages, CAF is well-documented for its interactions with dietary compounds, pharmaceuticals, and supplements, influencing cognitive function and physiological homeostasis. However, the potential pharmacokinetic and pharmacodynamic interactions between CAF—particularly from coffee, tea, and other commonly consumed beverages—and medications used in epilepsy management remain largely underestimated. Addressing this gap requires targeted research to define safe CAF intake thresholds that do not compromise the therapeutic efficacy of anticonvulsant drugs.

Thus, this study contributes to the growing body of research on the metabolic changes associated with epilepsy, particularly in the context of PTZ-induced seizures in zebrafish larvae. The findings underscore the utility of zebrafish as an intermediate model for epilepsy research, bridging *in vitro* studies and mammalian models. The high degree of genetic and physiological homology between zebrafish and humans supports the translatability of results, particularly in understanding seizure dynamics and metabolite alterations. Our metabolomic analysis revealed significant disruptions in lipid profiles, including increases in Lyso-PC, Lyso-PE, and Lyso-PAF in epileptic larvae. The influence of pharmacological agents such as CAF and TPM was also evident, particularly in modulating lipid metabolism. Notably, while CAF exacerbated lipid dysregulation, TPM showed a more stabilizing effect.

### 5.1 Study limitations

The studies discussed above show contradictory results, which may be a result of the use of different models referring to different epilepsy types, various methods for inducing seizures, differing methods of administering drugs, varying doses of chemical substances, and different measurement devices, acquirement methods, and parameters assessed. Moreover, we assessed metabolites in whole larvae rather than isolating and analyzing the brain specifically. This approach may dilute tissue-specific lipid signatures, particularly those originating from neurologically relevant regions such as the brain, thereby reducing the ability to detect subtle, region-specific alterations.

## Data Availability

The original contributions presented in the study are publicly available. This data can be found here: https://data.mendeley.com/datasets/j8cmn4bjxz/1.
